# Portable Quantification and Sustainable Active Packaging of Olive Pomace Polyphenols Obtained by Green Recovery

**DOI:** 10.3390/molecules31142476

**Published:** 2026-07-15

**Authors:** Natalia Gonzalez, Ezequiel Vidal, Carolina C. Acebal, Claudia E. Domini, Olivia V. López

**Affiliations:** 1 INQUISUR, Departamento de Química, Universidad Nacional del Sur, CONICET, UNS, Av. Alem 1253, Bahía Blanca 8000, Argentina; cacebal@uns.edu.ar (C.C.A.); cdomini@criba.edu.ar (C.E.D.); 2 Department of Chemistry and Biochemistry, California State University, San Marcos, CA 92078, USA; evidal@csusm.edu; 3 Planta Piloto de Ingeniería Química, PLAPIQUI (UNS-CONICET), Camino La Carrindanga Km 7, Bahía Blanca 8000, Argentina; olivialopez@plapiqui.edu.ar; 4 Departamento de Química, Universidad Nacional del Sur (UNS), Av. Alem 1253, Bahía Blanca 8000, Argentina

**Keywords:** olive pomace valorization, ultrasound-assisted extraction, polyphenols, portable 3D-printed device, active biobased packaging

## Abstract

This study explores the valorization of olive pomace through the green recovery of bioactive phenolic compounds for application in active packaging for olive oil preservation, alongside the development of a low-cost analytical strategy aligned with white analytical chemistry principles. Ultrasound-assisted extraction using 50% (*v*/*v*) aqueous ethanol significantly improved polyphenol recovery, reducing extraction time to 2 min while increasing efficiency compared to conventional maceration. Total phenolic content was determined using the Folin–Ciocalteu method and measured with both a UV–Vis spectrophotometer and a portable 3D-printed smartphone-based device, which showed excellent agreement with the reference method and comparable analytical performance. Optimized extracts were incorporated into starch–glycerol films, enhancing UV-barrier properties and enabling controlled release of phenolics. When applied to olive oil packaging, the films reduced color degradation under accelerated aging, indicating improved photo-oxidative stability. Composting tests suggested the biodegradation capability of the developed materials under the evaluated conditions. Overall, the study demonstrates an integrated sustainable approach combining waste valorization, active packaging development, and accessible analytical innovation.

## 1. Introduction

The olive oil industry represents an important sector of the global agri-food economy due to the growing demand for extra virgin olive oil and its recognized health-promoting properties, mainly associated with its phenolic compounds [1,2,3,4,5]. At the same time, olive oil production generates large quantities of by-products, particularly olive pomace, which still contain significant amounts of phenolic compounds and other valuable bioactive molecules. However, olive oil is highly susceptible to oxidative degradation during storage and commercialization, particularly when exposed to light, oxygen, and heat, leading to quality deterioration and loss of bioactive compounds. In this context, the development of sustainable strategies capable of preserving olive oil stability while simultaneously valorizing olive industry by-products has attracted increasing scientific interest.

Olive oil storage is a critical post-processing stage that must be optimized to minimize the oxidation of bio-active compounds, particularly phenolic compounds, which play a key role in oil stability and quality. Several strategies have been proposed to improve oxidative stability, including the direct incorporation of antioxidant compounds into olive oil. Markhali & Teixeira [6] evaluated the oxidative stability of extra virgin olive oil enriched with olive leaf extracts, reporting improved oleuropein retention compared to unfortified oils. Similarly, the enrichment of olive oil with natural antioxidants derived from tomato residues, bergamot fruits, and thyme-derived phenolic compounds has also been investigated [7,8,9].

Beyond the direct addition of antioxidants to olive oil, increasing attention has been devoted to the development of active packaging systems capable of controlling oxidation during storage. In particular, packaging materials incorporating natural antioxidant compounds have emerged as promising alternatives for extending the shelf life of oxidation-sensitive foods. Kurek et al. [10] developed chitosan/fish gelatin-based packaging films containing gallic acid as an antioxidant agent, while Rahman, Batsh, Gurumayam, Borah & Chowdhury [11] reported sodium alginate/nanocellulose bioactive films incorporating aqueous extracts of Moringa oleifera leaves for edible oil packaging applications. Likewise, de Rezende et al. [12] proposed κ-carrageenan films enriched with curcumin dispersions to improve the stability of packaged oils. These studies demonstrate the growing interest in sustainable antioxidant systems for oil preservation; however, the integration of agro-industrial waste valorization, active packaging development, and accessible analytical monitoring strategies remains limited.

Regarding this, the recovery of bioactive compounds from food by-products has received considerable attention due to the possibility of obtaining value-added ingredients for food, nutraceutical, and packaging applications. Extraction methods for phenolic compounds have evolved from conventional techniques such as maceration to innovative methodologies designed to preserve their bioactivity, where extraction yield, extract quality, and final effectiveness depend on the procedure employed [13]. In this context, the concept of green analytical chemistry (GAC) and white analytical chemistry (WAC) have promoted the development of extraction processes with reduced solvent consumption, low energy requirements, and minimized environmental impact. Among the emerging techniques for extracting bioactive compounds from agro-industrial biowaste, microwave-assisted extraction (MAE), supercritical fluid extraction (SFE), and ultrasound-assisted extraction (UAE) stand out [13,14,15]. UAE is based on the propagation of high-frequency ultrasonic waves that generate cavitation phenomena in the extraction medium, producing the formation and collapse of microbubbles that disrupt plant cell walls and enhance mass transfer. In terms of extraction yield, solvent consumption, and time efficiency, UAE outperforms conventional methods, making it a promising strategy for sustainable extraction processes [16,17].

Several analytical methods are currently available for the determination of phenolic compounds, ranging from spectrophotometric techniques for total polyphenol quantification to chromatographic methods capable of identifying individual phenolic species [18]. Among them, the Folin–Ciocalteu assay remains one of the most widely used methods for total phenolic content determination due to its simplicity [19]. Briefly, phenolic compounds reduce the Folin–Ciocalteu reagent in a basic medium, producing a colored complex that is subsequently quantified by molecular absorption spectrophotometry. Despite their analytical reliability, conventional benchtop UV–Vis spectrophotometers present several limitations related to portability, equipment cost, energy consumption, and sample/reagent requirements, which conflict with the principles of GAC, including miniaturization, energy efficiency, and waste reduction. Consequently, the development of portable analytical tools capable of providing reliable measurements while reducing equipment costs and laboratory infrastructure requirements is essential. In this context, smartphone-assisted analytical systems combined with 3D-printed devices have emerged as promising low-cost and sustainable alternatives aligned with WAC principles [20,21]. The continuous improvements in 3D-printing technology have provided analytical chemists with new opportunities to fabricate low-cost and accessible laboratory equipment, accessories, and instrumentation, which in many cases are comparable to those offered by specialized manufacturers. Furthermore, the widespread use of smartphone cameras for colorimetric measurements has stimulated the development of portable analytical systems due to their accessibility, ease of operation, and suitability for point-of-need analysis [21,22].

This study proposes an integrated strategy for the valorization of olive pomace within a circular economy framework. Ultrasound-assisted extraction was employed as a green and efficient methodology to recover high-value bioactive compounds from the pomace, aiming to surpass the yield of conventional methods. In parallel, total polyphenols were quantified using both conventional UV–Vis spectrophotometry and a portable 3D-printed smartphone-assisted analytical device, aiming to evaluate a low-cost and miniaturized alternative for accessible analytical measurements. Finally, the optimal extract obtained was incorporated into starch films to develop active bio-packaging specifically designed for single-serving portions of olive oil. This integrated approach combines agro-industrial waste valorization, sustainable extraction technologies, accessible analytical methodologies, and the development of antioxidant active packaging materials for the preservation of oxidation-sensitive foods.

## 2. Materials and Methods

### 2.1. Sample Preparation

Olive pomace, obtained as a by-product of the extraction of Coratina variety extra virgin olive oil from two consecutive years of production (2023–2024), was kindly supplied by Estilo Oliva (Finca Don Nicolás, Coronel Dorrego, Buenos Aires, Argentina). Samples were collected between March and April directly at the production site and frozen until their processing. The olive pomace was dried in a vacuum oven (Cole Parmer, Vernon Hills, IL, USA) at 60 °C until a constant weight was reached. Subsequently, the olive pomace samples were freeze-dried using a freeze dryer (LA-B3-C, Rificor, Buenos Aires, Argentina). Freeze-drying was performed under the operating conditions recommended by the manufacturer (−50 °C condenser temperature, 0.1 mbar vacuum, 48 h). Finally, they were crushed and sieved (420-micron mesh). A portion of these samples was defatted by Soxhlet extraction [23]. The resulting samples were classified as fresh pomace (O_F_), freeze-dried pomace (O_FD_), and defatted pomace (O_D_). They were stored in the dark at 20 °C until use. The moisture values, fat content, and FTIR spectra of the samples were reported in a previous work [24].

### 2.2. Optimization of the Polyphenol Extraction Method

Maceration and UAE optimization assays were independently performed on fresh (O_F_), freeze-dried (O_FD_), and defatted (O_D_) olive pomace samples. Then, 1.00 g of the sample was used to obtain the extracts (solute-to-solvent ratio of 1:10 *m*/*v*), the resulting liquid was filtered and stored at 4 °C for subsequent analysis. A one-way design was employed to evaluate the effects of key factors on the total phenolic content (TPC) to identify the ideal conditions for extracting the bioactive compounds. One of the variables studied was the ethanol content in the extraction solvent. Hydroethanolic solutions containing 40, 50, and 60% (*v*/*v*) ethanol (Porta, Buenos Aires, Argentina) were evaluated during the optimization process. For the extraction of phenolic compounds by maceration, maceration time (15–120 min) was also evaluated, as reported in the literature [14]. The optimized procedure was performed using a univariate approach by independently evaluating each experimental variable while keeping the remaining parameters constant.

For UAE, the Sonics Vibra-Cell VCX130 system ((Vibra-Cell VCX130, Sonics & Materials, Inc., Newtown, CT, USA) with a titanium probe (Ø = 9.5 mm) was used. The remaining variables were ultrasonic amplitude (60–100%) and ultrasonic time (1–5 min). The ultrasonic temperature was continuously monitored using a digital thermometer and maintained between 25 and 30 °C, while operating at 80% of the maximum nominal power (120 W).

All parameters were studied in triplicate.

### 2.3. Determination of Total Polyphenol Content of Olive Pomace Extract

The extracts were centrifuged and filtered using a 0.45 µm syringe filter (Millipore Sigma, Burlington, MA, USA). Then, an aliquot was taken to determine the TPC using the Folin–Ciocalteu method with several modifications [25]. Although this assay is widely used for the estimation of total phenolic content (TPC) in plant-derived extracts, it is important to note that the Folin–Ciocalteu reagent may also react with other reducing substances present in the samples. Previous characterization by HPLC of Coratina varietal olive pomace confirmed the presence of phenolic compounds [4]. The reaction mixture consisted of 500 μL of the sample extract, 12.50 mL of distilled water, and 1.25 mL of Folin–Ciocalteu reagent (Anedra, Buenos Aires, Argentina). After 4 min, 5.00 mL of sodium carbonate solution (0.20 g mL^−1^, Anedra, Buenos Aires, Argentina) was added, and the mixture was diluted to a final volume of 25.00 mL. After a 30 min reaction at room temperature, the TPC was quantified by UV–Vis spectrophotometry and by the portable 3D-printed device attached to a smartphone that was designed in the laboratory. Calibration curves were constructed using a standard gallic acid solution, GAE (Sigma–Aldrich, St. Louis, MO, USA), at different concentrations, expressed as mg gallic acid equivalents per gram of sample (mg GAE g^−1^ sample) (number of standard solutions, *N* = 5, number of replicates, *n* = 3). The data reflected in these calibration curves (obtained spectrophotometrically and with the portable 3D-printed device) were used in the quantification of TPC. The calibration curves and the corresponding analytical performance parameters are discussed in Section 3.2.

In the case of using UV–Vis spectrophotometry, the absorbance of the extracts was measured at 765 nm using a UV–Vis spectrophotometer Agilent 8453 (Agilent Technologies, Santa Clara, CA, USA).

The portable 3D-printed device developed in this study is described in the following section.

Portable 3D-Printed Device

- Instrument Description

A simple, portable, and reliable absorbance meter was designed and manufactured using FDM (Fused Deposition Modeling) 3D printing. This instrument enables fast, reliable, and reproducible field optical measurements based on image acquisition using a mobile phone. The proposed device features a linear optical geometry that uses a white LED (Light-Emitting Diode) as the radiation source (positioned 180° from the phone’s camera). The configuration also integrates a sample holder for a standard 10 mm PMMA (polymethyl methacrylate) cuvette. The light transmitted through the cuvette is directly captured by the smartphone image sensor, allowing for quantitative absorbance analysis by comparing sample and reference transmitted intensities. Once attached to the phone casing, the device requires no additional optical alignment, simplifying operation and ensuring measurement reproducibility.

- Design and manufacturing

**Figure 1:**
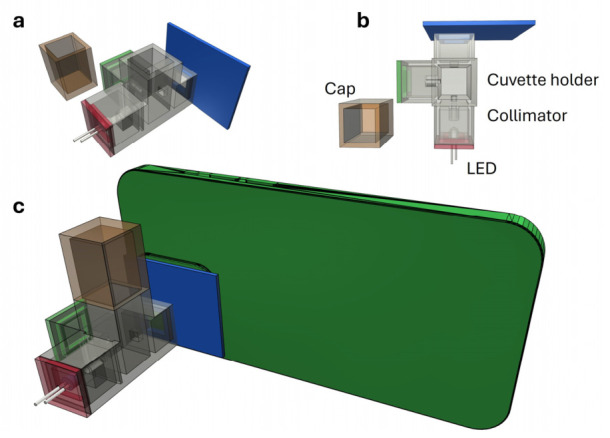
Portable 3D-printed device: (**a**) side view, (**b**) top view, and (**c**) portable 3D-printed device attached to a smartphone.

The device consists of four printed components (Figure 1): (i) a mounting plate for a standard smartphone protective case, (ii) a central body housing the optical system with collimators and cuvette holders, (iii) a cover for the white LED, and (iv) a closing cap that protects the cuvette camera and completely eliminates ambient light during capture. All components are fabricated from matte black polylactic acid (PLA) filament (Bambulab, Shenzhen, China) to protect the system from external light.

The assembled instrument has overall dimensions of 51.46 mm (length) × 42.00 mm (width) × 35.24 mm (height). These dimensions simplify handling the device once connected to the phone. The mounting plate (blue component in the design renderings) includes an opening to align the smartphone’s camera, ensuring the sensor is oriented toward the optical tunnel entrance (Figure 1). This mounting plate is permanently attached to a 3D-printed housing using cyanoacrylate adhesive. The printed parts are joined by click-fit connections without the need for additional adhesive. After this initial installation, no further alignment procedures are required: the camera, sensor, and LED remain collinear.

The central body of the device includes a holder compatible with standard spectrophotometric cuvettes and a removable top cup that prevents ambient light interference during acquisition (yellow section in Figure 1). Adjacent to the cuvette chamber, a 5 mm white LED directed toward the cuvette serves as the light source. A thin diffuser (80–100 μm tracing paper), positioned a few millimeters from the LED, homogenizes the emitted light and improves illumination uniformity due to the short LED–cuvette distance.

A narrow tunnel with a 1.2 mm opening was positioned between the cuvette and the camera to minimize stray light, allowing the detector to receive mainly the light transmitted through the cuvette. This configuration generated an illuminated circular region within a dark background, simplifying image processing. Thus, all the light detected originated exclusively from the LED–cuvette–camera optical path.

- Optical configuration

The system uses a 5 mm white LED powered by a 5 V USB-C adapter as the light source. The emitted light passes through a diffuser, the sample cuvette, and a collimation tunnel before being recorded by the smartphone rear camera. ISO and shutter speed were manually adjusted under open-shutter conditions and kept constant throughout the measurements to avoid automatic exposure variations. In addition, the fixed alignment between the camera and cuvette ensured a consistent region of interest and reduced user-related variability during image acquisition and processing.

- Operating procedure

To perform a measurement, the cuvette containing the blank, calibration standard, or sample was placed in the device, and the lid was closed to prevent external light interference. The LED was powered through the smartphone USB-C port, and images were acquired in manual mode using fixed ISO and shutter speed settings adjusted to avoid oversaturation. Three images were captured for each solution to minimize random noise and small variations in LED intensity. All images were subsequently transferred to a computer for processing.

- Image processing

Image analysis is performed using ImageJ v 1.52a. All images are loaded as a single stack to preserve their alignment and enable batch processing. A circular region of interest (ROI) is manually drawn around the illuminated area in a reference frame, and this same ROI is applied to the remaining images, as the bright region appears in the same position across the entire dataset.

For each frame, the average pixel intensity within the ROI was recorded for all three RGB (red, green, blue) channels. The analytical signal was obtained from the green channel, which exhibited the most consistent and proportional variation in intensity as light absorption increased. Transmittance values were calculated by comparing the image acquired without the cuvette, used as the incident light reference, with the transmitted light recorded for each sample. These values were subsequently converted into absorbance to construct the calibration curve. The green channel was selected because it provided the most stable signal and the best linear response throughout the evaluated concentration range, allowing for improved discrimination of the optical changes associated with the analytical measurements. Two processing approaches can be used. One option is to subtract the blank intensity to obtain absorbance-like values. The other is to use the raw intensities, which results in a negatively sloped calibration curve but still provides reliable quantitative results. In both cases, the relationship between transmitted intensity and concentration remains constant.

The blank-corrected values are normalized when necessary and plotted against the analyte concentration. A linear regression is then performed to obtain the slope, y-intercept, and R^2^ value. With this method, the processed intensities behave very similarly to absorbance values, even though no optical filters or wavelength selection components are involved. The information is extracted directly from digital images.

- Performance and validation

The analytical performance of the system—linearity, limits of detection, precision, and accuracy—was examined by comparing the signals obtained with the smartphone reader and the signals obtained with a conventional benchtop spectrophotometer.

### 2.4. Starch-Based Films Containing Extracts of Olive Pomace: Preparation and Characterization

Corn starch (St, Ingredion, Buenos Aires, Argentina) was used as the biopolymeric matrix and glycerol (G, Anedra, Argentina) as the plasticizer. Phenolic extracts were obtained using the optimized extraction method described previously from defatted olive pomace (O_D_) derived from the Coratina variety harvested in 2023 and 2024, yielding EO_D_2023 and EO_D_2024, respectively. Aqueous starch suspensions (5% *w*/*w*) were prepared and gelatinized in a thermostatic water bath at 90 °C for 20 min. The gelatinized suspensions were cooled to 40 °C and glycerol (1.5% *w*/*v*) and the phenolic extracts (10% *v*/*v*) were added. The suspensions were cast into polystyrene molds, maintaining a casting ratio of 0.5 g cm^−2^, and dried at 40 °C for 24 h. The evaluated formulations are shown in Table 1. The resulting films were removed and stored for subsequent evaluation of optical properties (color, transparency, opacity, and UV-barrier capacity).

Film color measurements were performed using a precision colorimeter NR110 (Shenzhen Linshang Technology Co., Ltd., Shenzhen, China) in transmittance mode. Color parameters L* (0–100), a* (negative values indicating greenness to positive values indicating redness), and b* (negative values indicating blueness to positive values indicating yellowness) were recorded according to the CIELab scale at least ten randomly selected positions on each film sample. Transparency, opacity, and UV-barrier capacity were determined from absorbance spectra (200–700 nm) recorded with an Agilent 8453 (Agilent Technologies, CA, USA) spectrophotometer. Film samples (4 cm × 0.9 cm) were placed on the inner face of a quartz spectrophotometer cell. Each sample was analyzed in triplicate. Film transparency was estimated following the procedure described by Han & Floros [26]. UV-barrier capacity was calculated according to the method reported by Castillo et al. [27], considering the area of the UV absorption peak and the film thickness.

The release of active compounds from the films containing extracts of olive pomace was evaluated through specific migration assays using a fatty food simulant, according to current recommendations for food-contact materials [28]. A 50% (*v*/*v*) aqueous ethanol solution was selected as a simulant of fatty foods due to its ability to mimic the polarity and extractive capacity of lipid-rich matrices. Film samples were cut into sections (approximately 4 cm^2^), immersed in 20.0 mL of the simulant and were maintained at room temperature. At predetermined time intervals (1, 2, 4, 6, 24, 48, 72, and 96 h), 500 μL aliquots of the simulant were collected for analysis. After each sampling, the same volume of fresh simulant was added to maintain a constant volume. The TPC released into the simulant was quantified by the Folin–Ciocalteu method using UV–Vis spectrophotometry and with the portable 3D-printed device attached to a smartphone, previously described.

### 2.5. Starch-Based Bio-Packaging Containing Olive Pomace Extracts for Individual Portions of Olive Oil

Bio-packaging (3 cm × 7 cm) was fabricated from the obtained films by heat sealing using an impulse-wire thermo-sealer. This sealing technique was chosen over the impulse bar since the first one allows the seal to be cooled before the jaws are opened, thereby enhancing its strength [29]. Obtained bio-packaging was subsequently filled with 4.0 mL of olive oil (Estilo Oliva, Coratina variety). The packaged oils were stored under UV irradiation to conduct an accelerated aging assay [30]. At predetermined storage intervals, samples of the packaged oils were collected, and their stability was assessed through colour variation using a precision colorimeter NR110 (Linshang, China), following the methodology described by Sikorska et al. [31]. Color measurements were performed in the CIELab space, recording L, a*, and b* parameters. In addition, the total color difference (ΔE) was calculated between day 0 and day 2 to quantify the overall change in color during storage (Equation (1)).

(1) $$∆E=\sqrt{{\left(L_{0}^{*}-L_{2}^{*}\right)}^{2}+{\left(a_{0}^{*}-a_{2}^{*}\right)}^{2}+{\left(b_{0}^{*}-b_{2}^{*}\right)}^{2}}$$

where L*_0_ and L*_2_ represent the lightness values of the oil at day 0 and day 2, respectively; a*_0_ and a*_2_ represent the chromatic coordinate along the green (−) to red (+) axis at day 0 and day 2; and b*_0_ and b*_2_ represent the chromatic coordinate along the blue (−) to yellow (+) axis at day 0 and day 2.

After the oil stability assays, the bio-packaging (BP-St-G, BP-St-G-EOD2023, and BP-St-G-EOD2024), corresponding to the previously prepared starch-based films after oil incorporation, were used to evaluate end-of-life composting. Prior to burial, each bio-packaging was placed inside an open-weave cloth bag to facilitate handling and recovery while allowing for free contact between the polymer surface and the composting microorganisms. Samples were buried in commercial special fertilized soil (Rincon Verde, Buenos Aires, Argentina) contained in individual plastic pots (volume 800 mL). The commercial special fertilized soil presented 18.5–21.5% organic matter, 49–51% ash content, 30.1% humidity, pH 6.5, and 0.5 mS cm^−1^ CE. Pots were maintained at 25 °C and 50% relative humidity throughout the assay. Pots were watered daily with approximately 50 mL water to maintain moisture suitable for aerobic composting. For each formulation (BP-St-G, BP-St-G-EO_D_2023, and BP-St-G-EO_D_2024) measurements were performed in triplicate (*n* = 3). Bio-packaging was retrieved at predetermined time points by removing the cloth bag from the compost, gently brushing off coarse compost particles and blotting with filter paper to remove excess moisture. Recovered samples were immediately weighed to obtain the wet recovered mass.

### 2.6. Statistical Analysis

The determinations were performed in triplicate (*n* = 3) and the results are reported as mean ± standard deviation. Statistical processing and visualization were performed using Origin 2018 (OriginLab, Northampton, MA, USA).

An analysis of variance (ANOVA) was performed to assess significant differences between sample groups, followed by Tukey’s post hoc test (*p* < 0.05) for multiple comparisons of means. Statistical significance was considered at a 95% confidence level (α = 0.05). All analyses were performed using the statistical software Infostat (version 2014).

## 3. Results and Discussion

### 3.1. Optimization of the Polyphenol’s Extraction Method

A comparative study was conducted to evaluate the extraction yield of total polyphenols from samples of O_F_, O_FD_, and O_D_, comparing the traditional maceration methodology with UAE, considered a green and sustainable technique [32]. Table 2 shows the evaluated parameters and optimum conditions that allow us to obtain the maximum TPC for both studied extraction methods. The optimization procedure was carried out independently for O_F_, O_FD_, and O_D_ samples, with comparable optimal conditions obtained for the three matrices. Figures S1 and S2 present the optimization results obtained independently for O_F_, O_FD_, and O_D_. Similar extraction trends were observed among the three matrices, resulting in common optimal extraction conditions (Table 2).

For both extraction methods, the optimum extraction solvent was ethanol 50% *v*/*v* in water. Particularly, in the case of maceration, the optimum maceration time was 30 min for all studied samples. Increasing the maceration time beyond 30 min did not yield a higher TPC. Maceration is the most widely used conventional method for extracting bioactive compounds because it is simple and economical, requiring only prolonged contact between the plant matrix and solvent, without specialized equipment. Furthermore, it is safe for heat-labile compounds, such as phenolic compounds. However, the use of ultrasound during extraction significantly reduced the extraction time to only 2 min.

### 3.2. Analytical Calibration and Performance

Polyphenol quantification was performed using a benchtop UV–Vis spectrophotometer and the portable 3D-printed device developed in the framework of this research. To compare the two devices, two calibration curves were constructed using standard solutions in the concentration range of 0.50–24.0 mg GAE L^−1^, prepared from a 200 mg L^−1^ gallic acid stock solution. The calibration curve obtained with the benchtop UV–Vis spectrophotometer showed a linear equation of y = 0.0248x + 0.0287 (R^2^ = 0.995), where x is the gallic acid concentration (mg L^−1^) and y is the absorbance. The limit of detection (LOD) was calculated from the calibration function, giving a value of 0.020 mg L^−1^, and the limit of quantification (LOQ) was calculated taking into account that LOQ = 10S_B_/b (where S_B_ is the standard deviation of the blank, and b is the slope of the calibration curve), yielding a result of 0.030 mg L^−1^.

**Figure 2:**
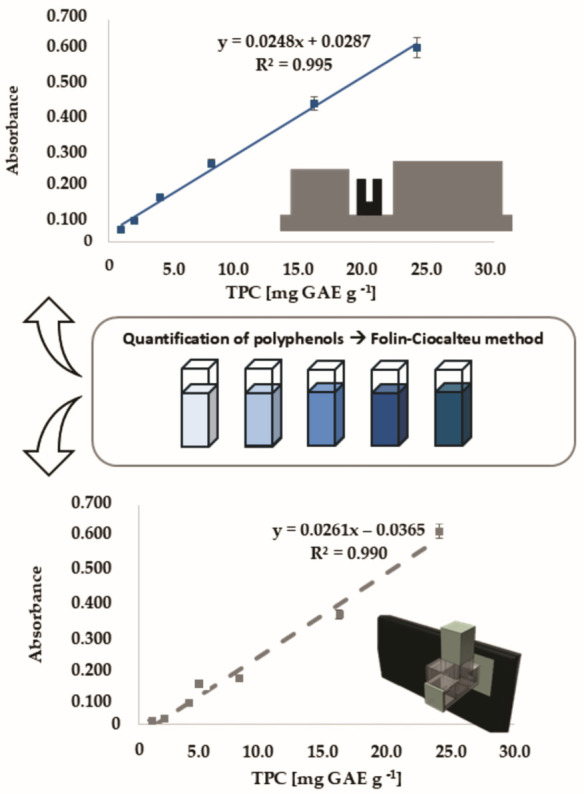
Calibration curves of the benchtop UV–Vis spectrophotometer (solid line) and of the portable 3D-printed device (dotted line).

In the case of using the portable 3D-printed device, the calibration curve for the GAE standard solutions was constructed using the green channel as the response (y = 0.0261x − 0.0365). The coefficient of determination, calculated from this calibration curve, was R_2_ = 0.990. Furthermore, the LOD and LOQ were 0.024 mg L^−1^ and 0.035 mg L^−1^, respectively. Compared to previously published methods, this approach provides improved limits of detection and quantification [33,34,35]. The relative standard deviation (%RSD) values were obtained from three independent determinations of each sample (parallel analysis). Intraday repeatability was assessed; the values obtained were less than 1.1% for all the standard solutions analyzed for both calibration curves. Inter-day precision was also evaluated using a 10 mg GAE L^−1^ standard solution analyzed over five different days (*n* = 7). The obtained %RSD was 3.0%, indicating acceptable intermediate precision and stable analytical performance of the proposed portable device over time. Figure 2 shows the calibration curves obtained for both methods.

In general, the values obtained with the portable 3D-printed device followed the same pattern as those of the spectrophotometer, and both methods agreed across the entire tested range. These observations indicate that the device can provide stable and reliable measurements despite its very simple hardware.

### 3.3. Determination of the Total Polyphenol Content

This section aims to evaluate the TPC of the best extracts obtained previously, both by maceration and by UAE, analyzing samples O_F_, O_FD_ and O_D_. Extraction efficiency was assessed in terms of total polyphenol recovery, as global extraction yield may also include non-phenolic soluble constituents not associated with the targeted bioactive fraction. As shown in Table 3, UAE yielded significantly higher TPC compared to maceration. Maceration, despite being an extraction method for heat-labile compounds, such as polyphenols, has the disadvantage of requiring a long extraction time, being cumbersome, and, as observed in Table 3, resulting in a lower TPC yield. Conversely, UAE takes advantage of the destructive forces generated by ultrasonic cavitation, such as shock waves and microjet formation, which effectively disrupt the studied samples. Polyphenols are then released from the plant matrix because of this disruption, which also facilitates solvent penetration [36]. Considering the context of a circular economy, it is important to mention the application of environmentally friendly extraction methods [37]. Therefore, it can be concluded that ultrasonic extraction using a hydroalcoholic solvent constitutes an effective approach for optimizing polyphenol recovery from the samples studied, combining short extraction times, reduced solvent requirements, and efficient recovery of phenolic compounds.

It is important to note that the Folin–Ciocalteu assay provides an estimation of the total reducing capacity commonly associated with phenolic compounds, although the reagent may also react with other reducing substances extracted from olive pomace. Therefore, the results obtained in this study should be considered as an estimation of the overall reducing compounds associated with phenolic constituents, rather than a precise quantification of individual phenolic molecules. Nevertheless, previous work included HPLC characterization of the polyphenol profile of olive pomace from the Coratina variety, confirming the presence of several phenolic compounds [4]. These findings support the interpretation that phenolic constituents contribute significantly to the higher Folin–Ciocalteu response observed after UAE. Accordingly, the higher values obtained for UAE suggest an enhanced recovery of reducing compounds associated with phenolic constituents from the olive pomace matrix.

To verify the veracity of the portable 3D-printed device developed within the framework of this work (proposed method), polyphenols were quantified against traditional quantification using benchtop UV–Vis spectrophotometry (reference method) [19,20,21,22,23,24,25]. Since the samples contain different amounts of TPC, a paired *t*-test was performed to compare the mean differences between data points. This paired *t*-test posits as its null hypothesis that there is no significant difference in the concentrations obtained by the two methods. Table 3 also included the statistical parameters obtained. The calculated t-values of 0.25 and 0.08 corresponding to maceration and UAE, respectively, are less than the critical t-value of 4.30 (α = 0.05; *n* − 1 = 3). It can be concluded that there were no significant differences between the two methods analyzed, using this confidence level.

#### 3.3.1. Method Validation and Comparison

Although the linearity and analytical merit for the proposed device proved to be consistent, the results obtained must be validated. The evaluation of systematic errors and compatibility of results must be ensured using different statistical tools. Therefore, the portable 3D-printed device results were validated using two different approaches against a spectrophotometric method.

**Figure 3:**
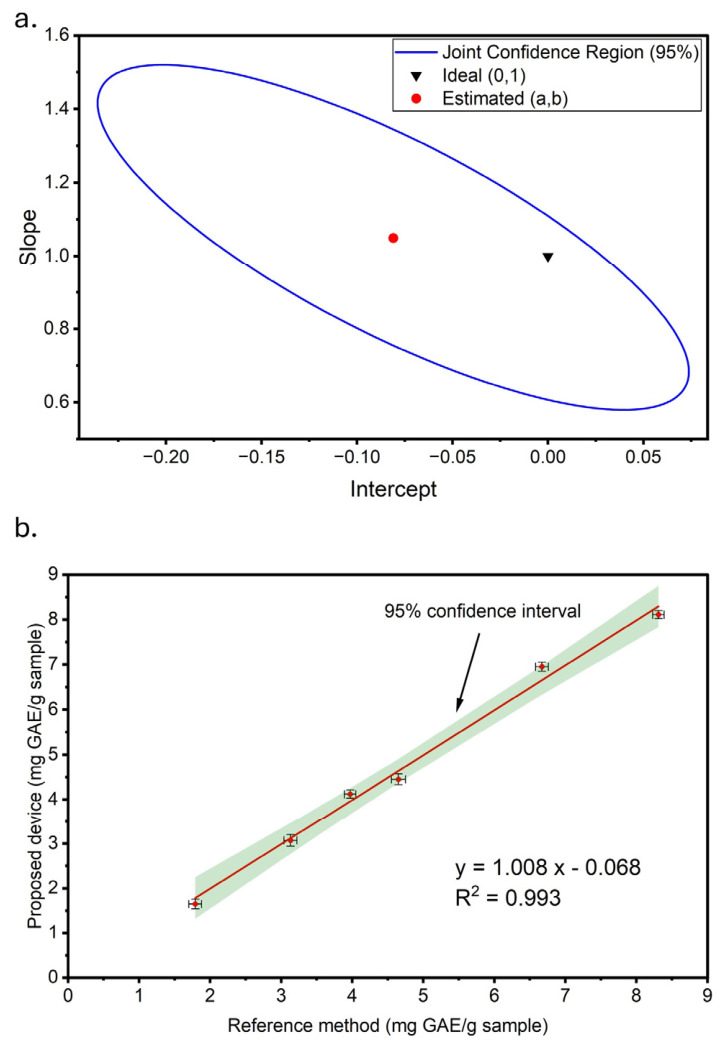
Validation of the proposed device: (**a**) Joint confidence interval test for slope and intercept showing the 95% confidence region and the theoretical point of ideal agreement and (**b**) method comparison using reference and proposed method in real samples and the experimental linear regression curve.

Firstly, paired absorbance values were compared using bivariate least-squares regression. The agreement between the methods was evaluated using the joint confidence interval test for the slope and the intercept (Figure 3a). The assessment showed a straight regression line: [proposed device] = 1.05 [reference method] − 0.08, showing a slope close to one and an intercept close to zero. These results suggest good agreement between the two methods.

The elliptical region represents 95% joint confidence for the covariance between slope and intercept estimates. Inside the ellipse area, any result would be considered consistent with experimental data. The point inside the ellipse corresponds to a slope equal to 1 and an intercept equal to zero (ideal result). As the abovementioned point appears inside the ellipse, there is no significant statistical evidence of bias, neither constant nor proportional between methods.

A second validation strategy was also applied. Real samples were analyzed using reference and proposed instruments. The samples were measured in triplicate. Mean values for each sample obtained with the portable 3D-printed device were plotted against those obtained with a spectrophotometer. The results shown in Figure 3b present a linear relationship with a slope of 1.01 and a determination coefficient (R^2^) of 0.993. The result suggests a good correlation between both methods and consistency across the proposed linear range. The previously determined LOD and LOQ values further supported the suitability of the proposed portable method for GAE quantification.

#### 3.3.2. Advantages and Applications

This device was built on earlier versions originally created for fluorescence measurements but includes several changes that make it suitable for absorbance measurements [38]. The optical layout is collinear, which allows the transmitted light to be measured directly without noticeable contributions from lateral scattering. The fixed alignment between the LED, the cuvette, and the camera also removes the need for optical calibration before each use. In addition, the 1.2 mm collimation tunnel and the matte black PLA interior help reduce stray light, which improves image contrast even when the measurements are taken under less controlled lighting conditions.

The instrument has a very low fabrication cost (below 2 USD), substantially lower than commercially available portable spectrophotometers and handheld colorimetric systems, which typically range from hundreds to thousands of USD depending on their analytical capabilities. In addition, this device weighs only a few grams (22.65 g), and up to ten complete instruments can be printed in a single run. These features make it practical for field applications, teaching laboratories, point-of-need measurements, and rapid environmental checks requiring simple, portable, and reliable equipment. Because the system uses a smartphone camera as a detector, it does not require extra electronics, microcontrollers, or optical benches. The materials are easy to obtain, and multiple units can be produced quickly.

This modular strategy is similar to what is often done in open-source, 3D-printed analytical tools, where additional functions can be incorporated later to meet the needs of different sample types or chemical systems.

### 3.4. Preparation and Optical Characterization of Starch-Based Films Containing Olive Pomace Extracts

**Figure 4:**
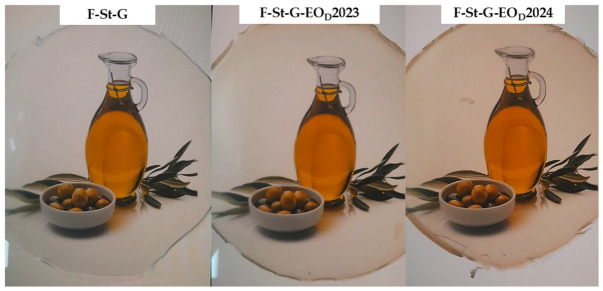
Starch-based films containing extracts of olive pomace.

The incorporation of olive pomace extracts into starch-based films was aimed at obtaining active materials rich in polyphenols with potential use in the packaging of foods susceptible to photo-oxidative deterioration. O_D_ extracts were chosen to be incorporated into starch-based films since they exhibited the highest total polyphenol content among the three olive pomace matrices evaluated (O_F_, O_FD_, and O_D_). The films obtained from the proposed formulations were easily removed from the molds and exhibited an excellent visual appearance (Figure 4).

Table 4 summarizes the chromaticity parameters of the studied films. The incorporation of olive pomace extracts into the film formulations led to noticeable changes in color, which can be mainly attributed to the characteristic hue of the agro-industrial by-product used.

Specifically, films containing olive pomace extracts showed significantly lower L* values and higher a* and b* values compared to the control formulation (F-St-G), indicating a reduction in lightness and a pronounced shift toward reddish–yellowish hues. These variations were more evident in films containing the EO_D_2024 extract, in agreement with their higher a* and b* values. These changes are consistent with the presence of phenolic compounds, pigments, and other chromophore substances naturally occurring in olive pomace, which are known to strongly influence the color properties of polymeric matrices. Similar trends have been reported for starch-based and other biopolymer films incorporating plant-derived extracts rich in polyphenols, flavonoids, and carotenoids [39,40]. The incorporation of polyphenol-rich natural extracts into biopolymer-based films commonly affects their optical properties, leading to darker coloration and increased opacity due to the intrinsic pigments and oxidation susceptibility of phenolic compounds [39].

**Figure 5:**
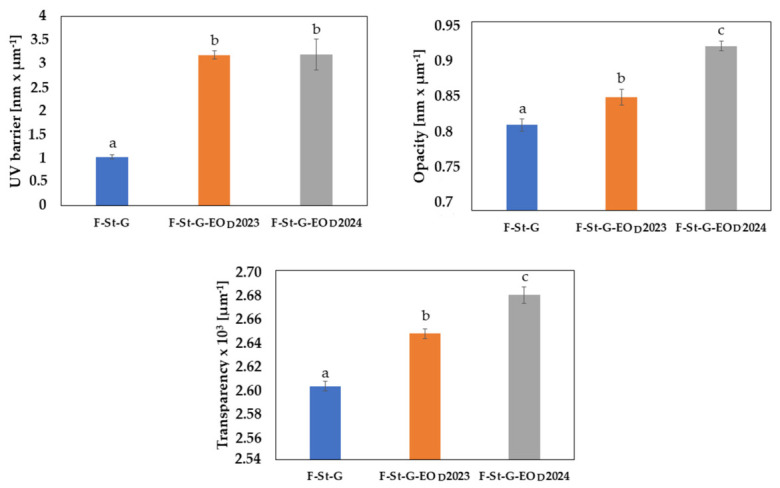
Optical properties of starch-based films containing extracts of olive pomace. Different letters indicate that there is a significant difference between samples by the Tukey test (*p* < 0.05).

Figure 5 shows the results corresponding to the optical properties of the developed films. Variations in opacity and transparency were observed because of extract incorporation. However, these changes were not excessively pronounced, and the films retained sufficient translucency to allow for adequate visualization of the packaged product. This behavior is desirable for food packaging applications, as it ensures consumer acceptance while still providing functional protection. Comparable results have been reported by other authors, who observed that the addition of plant or agro-industrial extracts to biodegradable films slightly decreases transparency, possibly due to associated with the presence of extract components incorporated into the polymer matrix [33,41]. Similar behavior was reported in other starch-based active films containing natural extracts, where the incorporation of phenolic-rich compounds caused moderate reductions in transparency and changes in coloration without compromising the visual appearance or applicability of the films as food packaging materials [41].

In addition, films containing olive pomace extracts exhibited a markedly higher UV-barrier capacity compared to the control films. This enhancement is likely related to the UV-absorbing ability of phenolic compounds present in olive pomace extracts, which act as natural light filters by absorbing radiation in the UV region. In agreement with these findings, Fotiadou et al. reported that chitosan/poly (vinyl alcohol) films enriched with olive pomace extract showed a significant reduction in UV transmittance [42]. Similar improvements in UV protection have also been described for starch-, gelatine-, and cellulose-based films containing olive-derived phenolics or other plant extracts [43,44,45,46,47]. Likewise, Nowak et al. [47] observed that the incorporation of selected plant extracts into gelatin and furcellaran-based double-layer films improved their UV-light barrier properties due to the presence of bioactive compounds capable of absorbing UV radiation.

The increased UV-barrier capacity of these films is particularly relevant for applications in the packaging of oxidation-sensitive foods, such as edible oils. Olive oil, in particular, is highly susceptible to photo-oxidative degradation due to its high content of unsaturated fatty acids and minor bioactive compounds. Therefore, the incorporation of olive pomace extracts into biodegradable starch-based films represents a promising strategy to enhance light protection while simultaneously valorizing agro-industrial by-products and potentially contributing to more resource-efficient packaging systems [48].

**Figure 6:**
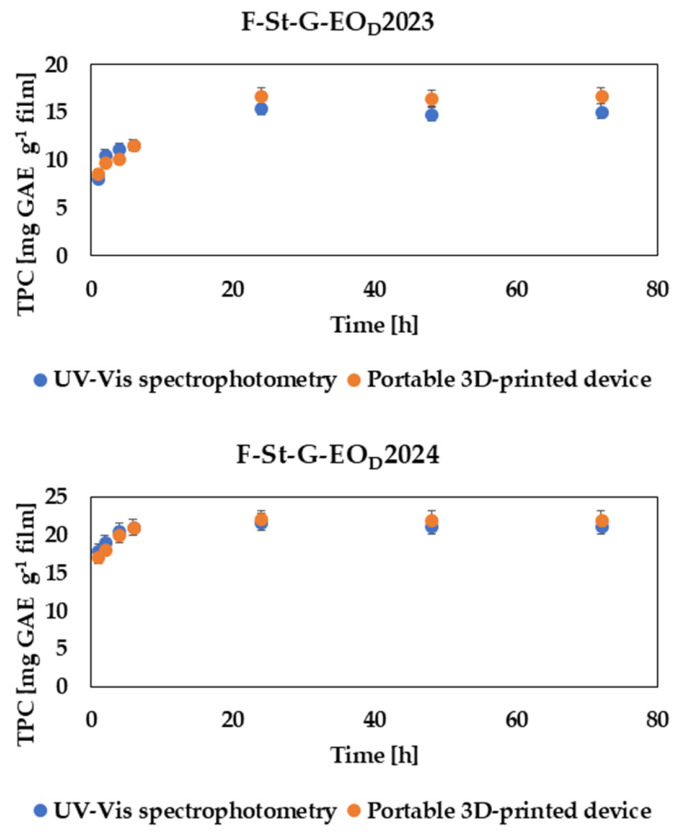
Release kinetics of phenolic compounds from starch–glycerol films containing olive pomace extracts into 50% (*v*/*v*) ethanol as a fatty food simulant.

Figure 6 shows the release kinetics of phenolic compounds from starch-based films containing olive pomace extracts into 50% ethanol, as quantified by using the reference and proposed methods. Both analytical methods exhibited comparable release profiles, confirming the reliability of the proposed analytical system.

The films exhibited a gradual, time-dependent release of phenolic compounds into the simulant, suggesting a diffusion-driven release behavior typical of hydrophilic polymer matrices. An initial release phase was observed during the first hours, followed by a slower stage to equilibrium. After 24 h, approximately 97% of the phenolic compounds incorporated into the films had migrated into the simulant, indicating an efficient and sustained release behavior. Similar release patterns have been reported for active films containing plant-derived polyphenols, where matrix characteristics and partial phenolic solubility favor diffusion into polar or semi-polar food simulants [49,50]. Comparable controlled release behavior was also described by Ramos et al. [50], who observed the progressive migration of carvacrol and thymol from polypropylene active packaging films, highlighting the importance of polymer active compound interactions and matrix characteristics in modulating release kinetics. The high release efficiency can be attributed to the swelling of glycerol-plasticized starch matrices in hydroalcoholic media, which enhances chain mobility and facilitates the diffusion of low-molecular-weight compounds, together with the predominantly free or weakly bound nature of olive pomace phenolics. The absence of a fast-release effect, combined with sustained migration over 24 h, suggests a controlled and sustained release behavior over the evaluated period, a desirable feature for active packaging applications requiring prolonged antioxidant activity.

Differences in release kinetics were observed between films containing extracts from two consecutive harvest years (F-St-G-EO_D_2023 and F-St-G-EO_D_2024). These variations can be attributed to year-to-year differences in the phenolic profile of olive pomace, which are influenced by agronomic practices, climatic conditions, and fruit maturity. Such compositional differences may affect the release behavior of phenolic compounds from starch-based films, leading to distinct release rates. Although 50% ethanol is widely accepted as a regulatory simulant for fatty foods and is frequently employed in migration studies due to its analytical convenience, it does not fully reproduce the physicochemical characteristics of real olive oil systems. Differences in polarity, viscosity, and solubilization capacity may affect the migration behavior of phenolic compounds and their partitioning between the packaging material and the food matrix. Therefore, the migration results obtained in this study should be interpreted as a preliminary estimation of phenolic release potential rather than a direct prediction of migration under actual olive oil storage conditions. Nevertheless, the assay provides a useful comparative approach for evaluating the release behavior of the polyphenols incorporated into the developed films under standardized conditions. Further studies using real olive oil systems would be valuable to confirm the release behavior of the polyphenols under practical application scenarios.

The migration assay provided a preliminary estimation of the behavior of the developed films under fatty food simulant conditions, supporting their potential applicability in olive oil packaging systems. Extra virgin olive oils contain relatively low phenolic levels, as most polyphenols remain in the pomace during oil extraction [51]. The controlled migration of phenolic antioxidants from the films may therefore help compensate for this depletion, enhancing oxidative stability and shelf life. Overall, the combination of efficient phenolic release and compostability supports the potential of these starch-based films as compostable active packaging materials for photo-oxidative-sensitive foods [52,53].

### 3.5. Starch-Based Bio-Packaging Containing Olive Pomace Extracts for Individual Portions of Olive Oil

**Figure 7:**
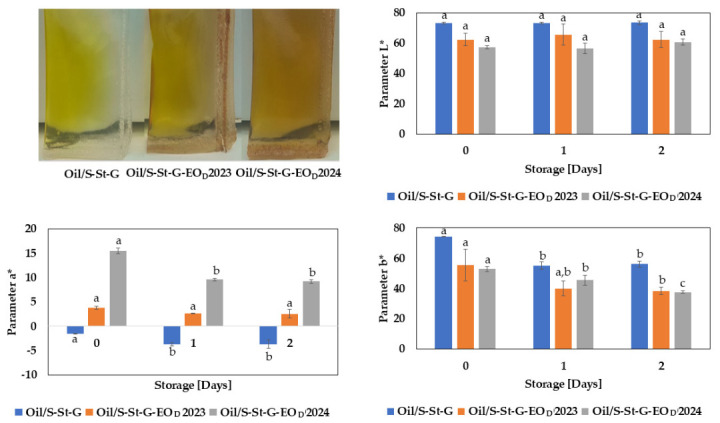
Bio-packaging of starch-based films containing extracts of olive pomace and the evolution of the CIELab color parameters of olive oil packaged in them during UV accelerated degradation. Different letters in the same series indicate that there is a significant difference between samples of the same formulation at different storage times by the Tukey test (*p* < 0.05).

Figure 7 shows the appearance of the starch-based bio-packaging and the evolution of the color parameters of olive oil packaged in them during UV accelerated degradation. A significant color modification was observed in all samples after 2 days of irradiation, confirming that the applied conditions effectively promoted photo-oxidative degradation of the oil. Nevertheless, clear differences were observed depending on the bio-packaging formulation.

Although the individual L*, a*, and b* coordinates are presented to visualize the direction of chromatic changes, the overall extent of color degradation was comparatively evaluated through ΔE values. Olive oil packaged in extract-free starch–glycerol bio-packaging (BP-St-G) exhibited the highest overall color change during storage, as reflected by the highest ΔE value (18.23) between day 0 and day 2. In contrast, oils packaged in bio-packaging containing olive pomace extracts from both harvest years (BP-St-G-EO_D_2023 and BP-St-G-EO_D_2024) showed lower ΔE values (17.08 and 16.70, respectively), indicating improved chromatic stability under UV exposure. Although all samples underwent substantial color variation (ΔE ≈ 16–18), the reduction observed in extract-containing bio-packaging demonstrates a protective effect associated with the incorporation of olive pomace extracts into the film matrix [36].

Analysis of the individual CIELab coordinates supports these findings. Oils packaged in BP-St-G bio-packaging showed a pronounced decrease in lightness (L*) and larger shifts in a* and b* values, consistent with pigment degradation and photo-oxidative browning. In contrast, bio-packaging containing olive pomace extracts attenuated these changes, suggesting reduced photo-induced color degradation under the evaluated conditions, which are particularly sensitive to light-induced oxidation.

The improved color stability observed in extract-containing bio-packaging may be associated with the antioxidant properties of phenolic compounds present in olive pomace extracts present in olive pomace. These compounds are known to act as radical scavengers and metal chelators, thereby inhibiting oxidative reactions. When incorporated into biodegradable films, these compounds may contribute to reducing oxidation-related changes through antioxidant activity and UV-screening effects [54].

In addition to their chemical antioxidant action, olive pomace extracts have been reported to enhance the UV-barrier properties of biodegradable films. This physical effect limits the transmission of high-energy UV radiation that accelerates photodegradation of oil pigments. The combination of antioxidant activity and improved UV screening has been previously described for active packaging films containing olive-derived by-products and provides a coherent explanation for the reduced color changes observed in the present study [37].

Considering commonly accepted CIELab thresholds, ΔE values greater than 5 correspond to clearly perceptible color differences. Therefore, the ΔE magnitudes measured here indicate pronounced color alteration under the severe conditions of the accelerated test. However, the relative reduction in ΔE for bio-packaging containing olive pomace extracts highlights their capacity to mitigate deterioration under identical stress conditions, emphasizing the relevance of comparative ΔE analysis when evaluating active packaging performance [55].

Overall, these results demonstrate that starch-based bio-packaging enriched with olive pomace extracts confers measurable protection to olive oil packaged in individual portions under UV accelerated aging. This protection arises from the combined chemical and physical effects of the extracts and is consistent with previous reports on the use of olive by-products as functional additives in starch-based active packaging systems [37,49]. Although color preservation provided useful evidence of the protective effect of the developed bio-packaging against photo-induced deterioration, it represents an indirect indicator of oxidative stability. Therefore, future studies incorporating complementary oxidation parameters, such as peroxide value, p-anisidine value, and volatile oxidation markers, would provide a more comprehensive assessment of the antioxidant performance and protective efficacy of the active packaging system.

**Figure 8:**
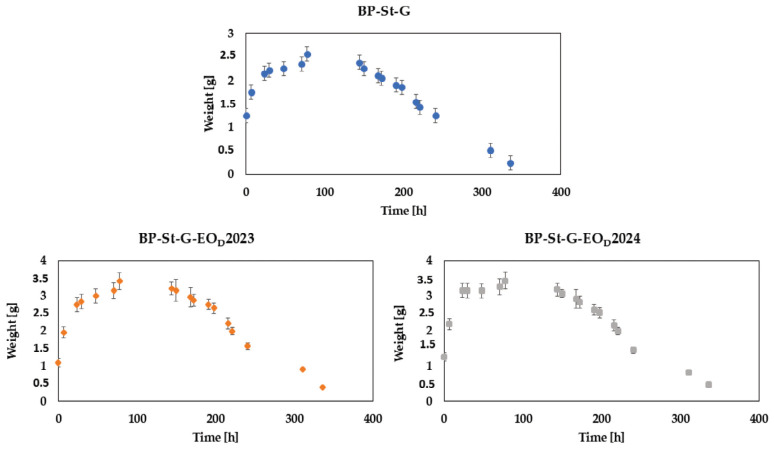
Mass evolution of sachets of starch-based films containing extracts of olive pomace during composting.

The evolution of the weight of bio-packaging as a function of composting was evaluated. All samples were buried in an organic compost after being emptied of their oil content, and weight measurements were taken at defined intervals (Figure 8). In all formulations an initial increase in mass was observed during the early composting period. This transient weight gain is characteristic of hydrophilic starch-based matrices plasticized with glycerol; the polymeric network rapidly absorbs moisture from the compost and undergoes reversible swelling before significant polymer chain scission occurs. The hygroscopicity of glycerol accelerates moisture uptake and swelling phenomena in starch films. Such behavior has been widely reported for starch-based films and is consistent with the known effects of plasticizers on water sorption and film morphology [56]. Following the initial swelling phase, a progressive decrease in sample mass was recorded for all formulations, consistent with hydrolytic depolymerization of starch followed by microbial assimilation of soluble fragments. The BP-St-G control films showed a slightly faster and more pronounced mass loss compared with the films containing olive pomace extracts, indicating a higher weight loss rate in the absence of added phenolic compounds. This trend aligns with previous composting studies showing that the more open and hydrophilic the starch matrix generally exhibits higher microbial accessibility and weight loss under aerobic composting conditions [57].

The BP-St-G-EO_D_ bio-packaging also underwent appreciable weight loss, confirming its compostability after practical use as oil bio-packaging. However, the degradation kinetics were modulated by the presence of phenolic compounds, as the phenolic-containing films tended to retain more mass than the control at several composting times. Two mechanisms may contribute to this effect. The slightly slower degradation observed for phenolic-containing films may be associated with changes in matrix organization and reduced microbial activity promoted by the presence of phenolic compounds. Second, polyphenols often exhibit antimicrobial properties that may transiently reduce the activity of certain compost microbes during the early stages of composting. The presence of phenolic compounds may have contributed to slightly slower composting kinetics during the initial assay stages. Figure S3 shows some photographs of the pots employed for composting assays of bio-packaging and of the samples after different composting times.

These roles of olive pomace phenolics in active packaging and their interaction with biodegradable polymer matrices have been recently documented [58]. The fact that both bio-packaging types degraded substantially under realistic composting conditions after use (i.e., containing residual oil) supports their practical suitability for inclusion in organic waste streams. Although residual lipids can sometimes affect microbial community composition and local oxygen diffusion in compost, in this study any such effects did not halt polymer composting. It should be noted that the composting assessment performed in this study was based primarily on mass-loss measurements and visual observations of material disintegration under controlled composting conditions. Standardized biodegradation indicators, such as CO_2_ evolution, oxygen consumption, or mineralization measurements according to ASTM or ISO protocols, were not evaluated. Therefore, the results should be interpreted as evidence of compostability and physical disintegration under the tested conditions rather than as a complete biodegradation assessment. Designing bioactive, compostable bio-packaging that balances antioxidant and antimicrobial functionality with acceptable end-of-life degradability is feasible and represents a promising approach for active food packaging applications [59].

## 4. Conclusions

This work demonstrates an integrated approach to olive pomace valorization through the recovery of bioactive phenolic compounds, their incorporation into compostable starch-based packaging materials, and the development of a low-cost analytical tool aligned with the principles of green analytical chemistry. Ultrasound-assisted extraction using 50% (*v*/*v*) hydroethanolic solution proved more efficient than conventional maceration, providing higher total polyphenol recovery with a significantly shorter extraction time.

The proposed portable 3D-printed device showed good agreement with conventional UV–Vis spectrophotometry, demonstrating its potential as an accessible and low-cost alternative for polyphenol determination. In addition, olive pomace extracts were successfully incorporated into starch-based films, improving UV-barrier properties and enabling controlled phenolic release into a fatty food simulant. Although migration studies provided valuable information regarding the release behavior of the developed films, they were conducted using a standardized food simulant rather than real olive oil systems. Therefore, further studies employing real food matrices would be valuable to confirm release performance under practical storage conditions. The resulting bio-packaging reduced color degradation of olive oil under accelerated UV aging and showed substantial mass loss and physical disintegration during composting under the evaluated conditions. However, further studies employing standardized biodegradation protocols are required to confirm the biodegradation performance of the developed films under controlled conditions. Although color preservation indicated a protective effect against photo-induced deterioration, future studies incorporating complementary oxidative stability indicators, such as peroxide value, p-anisidine value, and volatile oxidation markers, would provide a more comprehensive evaluation of packaging performance.

Overall, this study provides an integrated approach based on olive pomace valorization, low-cost analytical innovation, and functional packaging development, offering a promising route for the preservation of photo-oxidative-sensitive foods and the development of future food-packaging technologies.

## Figures and Tables

**Table 1:** Starch-based film formulations containing olive pomace extracts.

Film Formulation	Starch (St) [%, *w*/*w*]	Glycerol (G) [%, *w*/*v*]	Extract from Olive Pomace 2023 (EO_D_2023) [%, *v*/*v*]	Extract from Olive Pomace 2024 (EO_D_2024) [%, *v*/*v*]
F-St-G	5	1.5	0	0
F-St-G-EO_D_2023	5	1.5	10	0
F-St-G-EO_D_2024	5	1.5	0	10

**Table 2:** Evaluated parameters and optimum conditions of maceration and UAE for polyphenol extraction from olive pomace. Optimal conditions common to O_F_, O_FD_, and O_D_ samples.

Parameters	Type/Range	Optimal Conditions	
		Maceration	UAE
Extraction solvent ratio [EtOH % *v*/*v* in water]	40–60	50	50
Maceration time [min]	15–120	30	-
Ultrasound time [min]	1–5	-	2
Ultrasonic amplitude [%]	60–100	-	80

**Table 3:** Comparison of the results obtained in the extraction by maceration and UAE using the proposed method (portable 3D-printed device) and the reference method (spectrophotometric) for quantification.

Extraction Method	Sample	TPC Concentration [mg GAE g^−1^ Sample]		Difference Between Means	Statistical Parameters
		Proposed Method	Reference Method		
Maceration	O_F_	1.65 ± 0.11 ^a^	1.79 ± 0.09 ^a^	−0.14	x_d_ = −0.06
	O_FD_	4.12 ± 0.09 ^b^	3.97 ± 0.08 ^b^	0.15	s = 0.18
	O_D_	4.45 ± 0.12 ^c^	4.65 ± 0.10 ^c^	−0.2	t_c_ = −0.25
UAE	O_F_	3.07 ± 0.13 ^d^	3.13 ± 0.09 ^d^	−0.06	x_d_ = −0.02
	O_FD_	6.96 ± 0.10 ^e^	6.67 ± 0.09 ^e^	0.2	s = 0.20
	O_D_	8.11 ± 0.09 ^f^	8.31 ± 0.08 ^f^	−0.2	t_c_ = −0.08

x_d_: average of the differences; s: standard deviation of the differences; t_c_: t calculated for (*n* − 1) degrees of freedom. Different letters indicate a significant difference between samples by the Tukey test (*p <* 0.05). O_F_, fresh olive pomace; O_FD_, freeze-dried olive pomace; O_D_, defatted olive pomace; UAE, ultrasound-assisted extraction.

**Table 4:** Chromaticity parameters of starch-based films contain extracts of olive pomace.

Film Formulation	L*	a*	b*
F-St-G	88.77 ± 1.42 ^a^	−0.23 ± 0.01 ^a^	1.40 ± 0.09 ^a^
F-St-G-EO_D_2023	82.79 ± 2.76 ^b^	1.15 ± 0.12 ^b^	13.76 ± 0.77 ^b^
F-St-G-EO_D_2024	80.87 ± 0.40 ^b^	4.17 ± 0.13 ^c^	22.28 ± 0.37 ^c^

Different letters in the same column indicate that there is a significant difference between samples by the Tukey test (*p* < 0.05).

## Data Availability

The original contributions presented in this study are included in the article and Supplementary Materials. Further inquiries can be directed to the corresponding author.

## Supplementary Materials

The following supporting information can be downloaded at: https://www.mdpi.com/article/10.3390/molecules31142476/s1. Figure S1: Optimization of extraction time (a) and ethanol concentration (b) for maceration extraction of O_F,_ O_FD_, and O_D_ using ethanol as extraction solvent. The effects of extraction time and ethanol concentration on total polyphenol recovery are shown for each matrix; Figure S2: Optimization of ultrasound-assisted extraction conditions O_F_, O_FD_, and O_D_ using ethanol as extraction solvent. The effect of (a) ultrasonic amplitude, (b) ultrasound treatment time, and (c) ethanol concentration on total polyphenol recovery is shown for each matrix; Figure S3: Photographs of the pots employing for composting assays of bio-packaging and of the samples after different composting time.

## Abbreviations

The following abbreviations are used in this manuscript:

GAC	Green Analytical Chemistry
WAC	White Analytical Chemistry
MAE	Microwave-Assisted Extraction
SFE	Supercritical Fluid Extraction
UAE	Ultrasound-Assisted Extraction
O_F_	Fresh Pomace
O_FD_	Freeze-Dried Pomace
O_D_	Defatted Pomace
TPC	Total Phenolic Content
FDM	Fused Deposition Modeling
LED	Light Emitting Diode
PMMA	Polymethyl Methacrylate
PLA	Black Polylactic Acid
EO_D_2023	Extract from Olive Pomace 2023
EO_D_2024	Extract from Olive Pomace 2024
LOD	Limited of Detection
LOQ	Limit of Quantification
F-St-G	Starch-glycerol films
F-St-G-EO_D_2023	Starch-glycerol films with defatted phenolic extracts 2023
F-St-G-EO_D_2024	Starch-glycerol films with defatted phenolic extracts 2024
BP-St-G	Starch-glycerol Bio-packaging
BP-St-G-EO_D_2023	Starch-glycerol Bio-packaging with defatted phenolic extracts 2023
BP-St-G-EO_D_2024	Starch-glycerol Bio-packaging with defatted phenolic extracts 2024
