# Supplementary material for: Portable Quantification and Sustainable Active Packaging of Olive Pomace Polyphenols Obtained by Green Recovery
Source: Molecules. 2026 Jul 15;31(14):2476. doi: 10.3390/molecules31142476 (PMC13416019; doi:10.3390/molecules31142476)
Supplement: Supplementary file 1 [file molecules-31-02476-s001.zip › molecules-4341593-supplementary.pdf]

Portable Quantification and Sustainable Active Packaging of Olive Pomace Polyphenols Obtained by Green Recovery

Supplementary Information

Natalia Gonzalez,*[a] Ezequiel Vidal, [b] Carolina C. Acebal, [a] Claudia E. Domini, [a] and Olivia V. López, [c,d]

[a] INQUISUR, Departamento de Química, Universidad Nacional del Sur (UNS)-CONICET, Av. Alem 1253, 8000 Bahía Blanca, Argentina.

[b] Department of Chemistry and Biochemistry, California State University, San Marcos, California 92078, United States.

[c] Planta Piloto de Ingeniería Química (PLAPIQUI, UNS-CONICET), Camino La Carrindanga km. 7, Bahía Blanca, 8000, Buenos Aires, Argentina.

[d] Departamento de Química (Universidad Nacional del Sur, UNS), Av. Alem 1253, Bahía Blanca, 8000, Buenos Aires, Argentina.

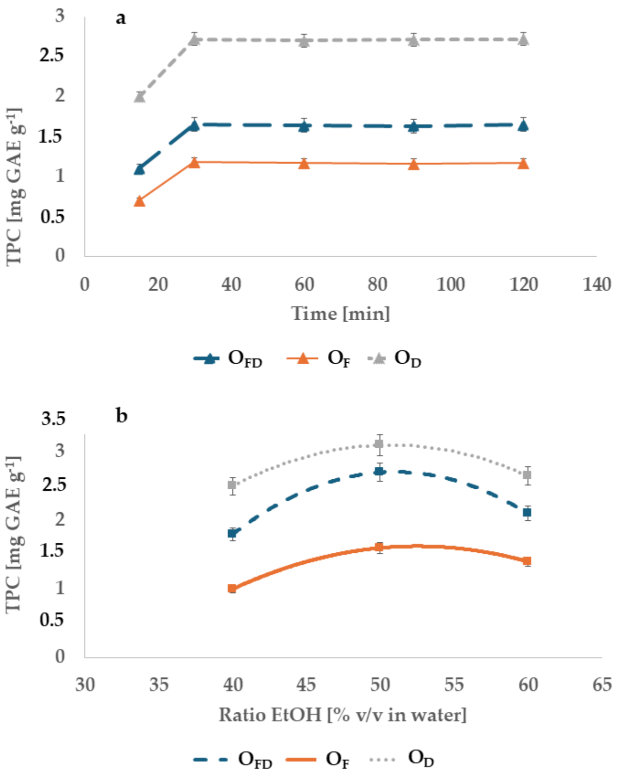

Figure S1. Optimization of extraction time (a) and ethanol concentration (b) for maceration extraction of O_F, O_{FD}, and O_D using ethanol as extraction solvent. The effects of extraction time and ethanol concentration on total polyphenol recovery are shown for each matrix.

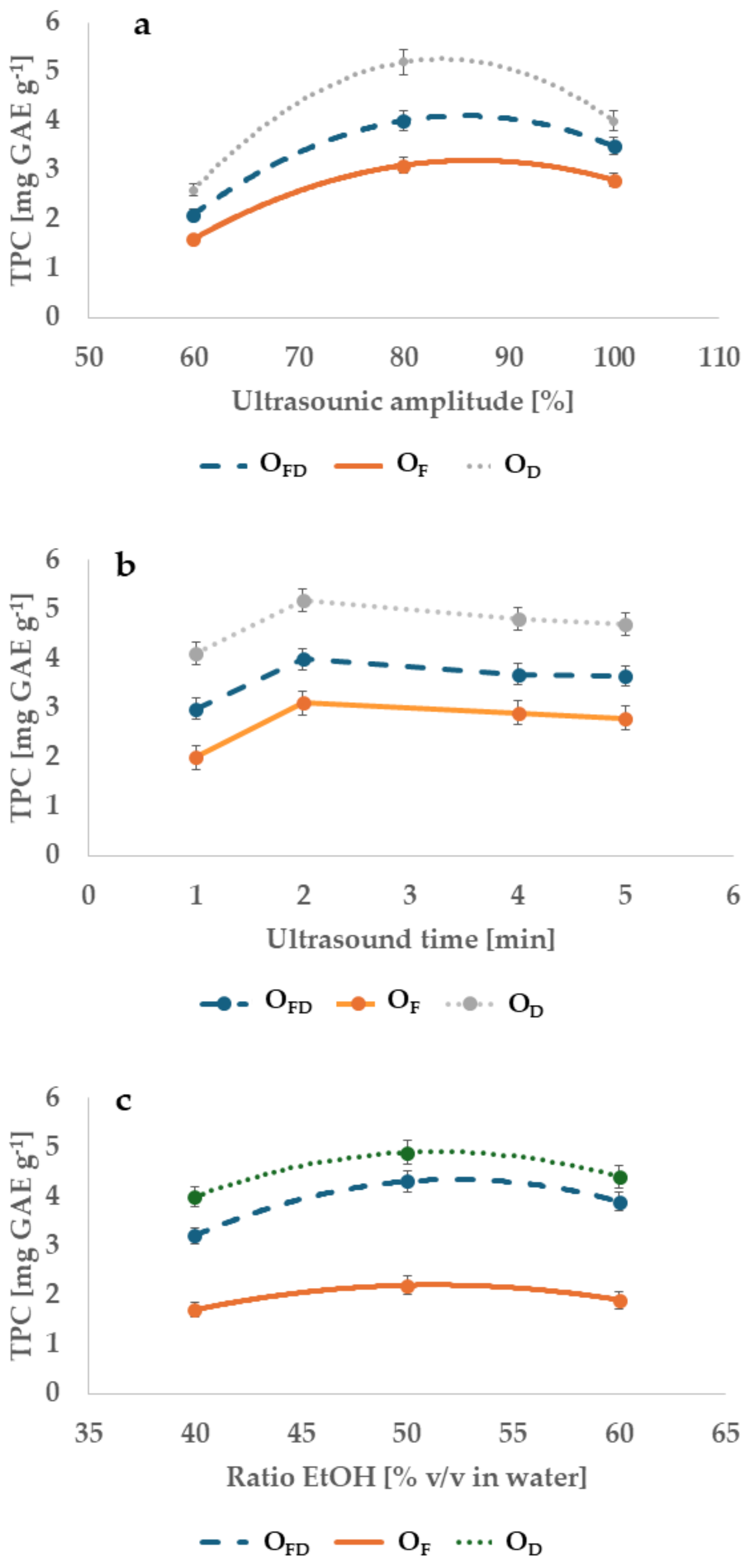

Figure S2. Optimization of ultrasound-assisted extraction conditions O_F, O_{FD}, and O_D using ethanol as extraction solvent. The effect of (a) ultrasonic amplitude, (b) ultrasound treatment time, and (c) ethanol concentration on total polyphenol recovery is shown for each matrix.

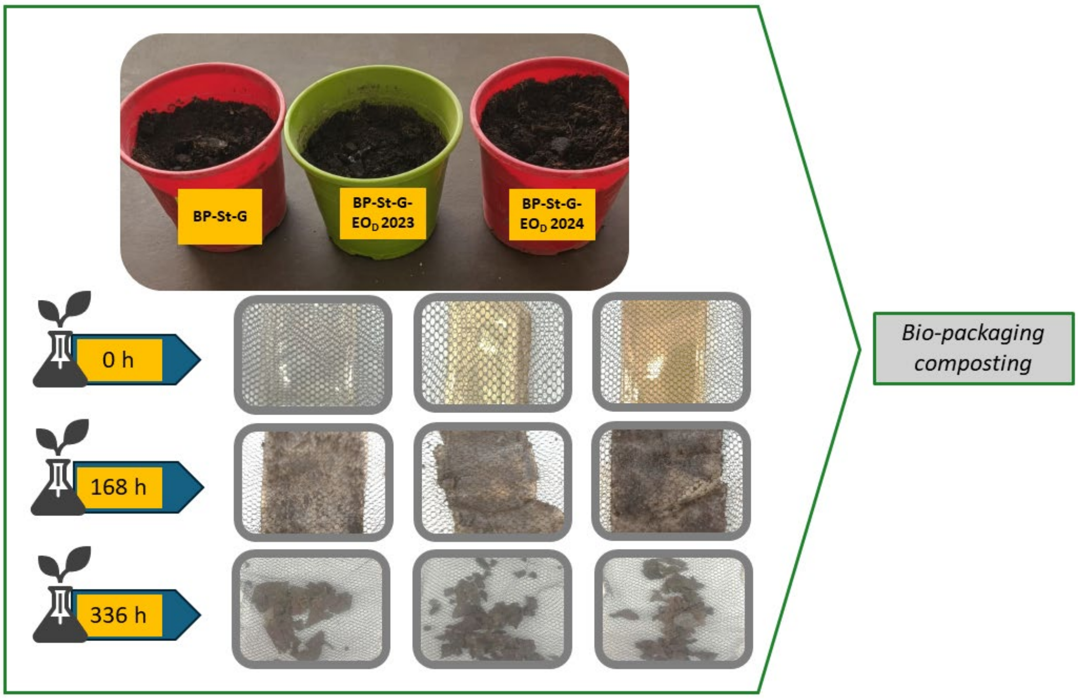

Figure S3. Photographs of the pots employing for composting assays of bio-packaging and of the samples after different composting time.
